# RNA-Sequencing Analyses Demonstrate the Involvement of Canonical Transient Receptor Potential Channels in Rat Tooth Germ Development

**DOI:** 10.3389/fphys.2017.00455

**Published:** 2017-06-29

**Authors:** Jun Yang, Wenping Cai, Xi Lu, Shangfeng Liu, Shouliang Zhao

**Affiliations:** Department of Stomatology, Huashan Hospital, Fudan UniversityShanghai, China

**Keywords:** tooth germ, tooth development, RNA-seq, canonical transient receptor potential channels (TRPC), odontogenesis

## Abstract

Tooth development depends on multiple molecular interactions between the dental epithelium and mesenchyme, which are derived from ectodermal and ectomesenchymal cells, respectively. We report on a systematic RNA sequencing analysis of transcriptional expression levels from the bud to hard tissue formation stages of rat tooth germ development. We found that *GNAO1, ENO1, EFNB1, CALM1, SIAH2, ATP6V0A1, KDELR2, GTPBP1, POLR2C, SORT1*, and members of the canonical transient receptor potential (*TRPC*) channel family are involved in tooth germ development. Furthermore, Cell Counting Kit 8 (CCK8) and Transwell migration assays were performed to explore the effects of these differentially expressed genes (DEGs) on the proliferation and migration of dental pulp stem cells. Immunostaining revealed that TRPC channels are expressed at varying levels during odontogenesis. The identified genes represent novel candidates that are likely to be vital for rat tooth germ development. Together, the results provide a valuable resource to elucidate the gene regulatory mechanisms underlying mammalian tooth germ development.

## Introduction

Mammalian tooth development is modulated by sequential interactions between the oral epithelium and the underlying mesenchyme, which is analogous to the interactions observed in the development of other organs, such as the kidneys, glands, hair, etc. (Shiotsuka et al., 2013). A specific spatiotemporal molecular pattern of reciprocally inductive epithelium–mesenchyme interactions is the core mechanism underlying the development of the bud, cap, and bell stages (Koussoulakou et al., 2009; Kero et al., 2014). Various signaling molecules and transcription factors in the dental epithelial and mesenchymal tissues participate in these consecutive events. There are more than 300 genes associated with the formation and morphologic changes of tooth germs (Kihara et al., 2014). A better understanding of the signaling network is crucial to enhance our knowledge of tooth formation, regeneration, and potential treatments for tooth anomalies. However, the precise molecular mechanism underlying tooth germ development is still not fully understood.

The detailed study of gene expression profiles is complicated, and RNA sequencing (RNA-Seq) is currently the best approach for this type of study. RNA-Seq is a new and powerful deep-sequencing approach for studying gene expression and identifying novel RNA species. It can offer faster and more accurate measurements of transcriptional expression levels compared with other traditional means (Wang Z. et al., 2009). In recent years, because of the great reduction in time and cost, deep sequencing has been widely used in a variety of biological studies, including studies of pathogens, embryonic development, etc. (Kumar et al., 2016). It has been used in research on tooth germ development. For example, a recent investigation using RNA-Seq compared the gene expression profiles of mouse tooth germs under different *YAP* (a Hippo signaling transcriptional coactivator) gene knockout conditions (Liu et al., 2015).

In this study, we conducted an RNA-Seq analysis to assess gene expression during rat tooth germ development and we identified multiple differentially expressed genes (DEGs) associated with the different stages. The protein protein interactions (PPIs) of the proteins encoded by the DEGs were investigated and quantitative real-time polymerase chain reaction (qRT-PCR) was used to confirm the identified DEGs. We found that *GNAO1, ENO1, EFNB1, CALM1, SIAH2, ATP6V0A1, KDELR2, GTPBP1, POLR2C, SORT1*, and a novel family of canonical transient receptor potential (*TRPC*) channels were involved in rat tooth germ development. In addition, we explored the effects of inhibiting the expression of the above genes on rat dental pulp stem cells (DPSCs). The aim of our analysis was to reveal the molecular mechanisms underlying tooth germ progression and to provide clues regarding potential treatments for tooth anomalies.

## Materials and methods

### Tissue acquisition and preparation

Sprague Dawley (SD) rats (Department of Laboratory Animal Science, Fudan University, Shanghai, China) were used at the starting point in this study. Embryonic day 0 (E0) refers to the day on which the formation of a vaginal plug was identified. Embryos at stages E14.5, E16.5, and E18.5 and postnatal rats at 1 day (P1) and 7 days (P7) after birth were used. The first mandibular molar tooth germs were excised from the rats under a zoom stereo microscope (Olympus SZ51, Tokyo, Japan) and prepared for the following experiments. The rats were euthanized using phenobarbital sodium (i.p., 50 mg/kg) anesthesia. This study was approved by the Institutional Animal Care and Use Committee of Fudan University.

### RNA-Seq and PPI network analysis

To construct each cDNA library, the total cellular RNA was extracted from the tooth germ cells in the embryonic (E14.5, E16.5, and E18.5) and postnatal (P1 and P7) phases using Trizol reagent (Invitrogen, Carlsbad, CA, USA). For each stage, more than six tooth germs were used. The construction of each cDNA library was implemented following the manufacturer's suggestions and next-generation sequencing was carried out using an Illumina HiSeq 2000 platform (IGA, Udine, Italy). An RNA-Seq analysis was conducted to identify and visualize the expression of DEGs involved in tooth germ development. The bioinformatics analysis involved a heatmap analysis of DEGs based on the RNA-seq results.

Moreover, to further study the relationships among the DEGs at the protein level, we established the PPI network (based on the identified DEGs) by integrating protein information from the Search Tool for the Retrieval of Interacting Genes (STRING) database (http://string-db.org/). The Database for Annotation, Visualization and Integrated Discovery (DAVID) online platform (https://david.ncifcrf.gov/) was used to analyze the Gene Ontology (GO) and Kyoto Encyclopedia of Genes and Genomes (KEGG) categorizations of each of the DEGs.

### qRT-PCR

To validate the RNA-Seq data, we performed qRT-PCR to quantify the expression levels of candidate DEGs. As a positive control, we used the adenomatous polyposis coli (APC) gene, which is a Wnt signaling pathway inhibitor that plays a critical role in tooth development (Wang X. P. et al., 2009). Total RNA was extracted from tissues using standard methods that involved the use of phenol/chloroform with Trizol reagent. Subsequently, cDNA was synthesized using oligo- deoxythymine (dT) primers and reverse transcriptase (Superscript III; Invitrogen, Carlsbad, CA, USA). After cDNA synthesis, qRT-PCR was performed using sequence-specific primer pairs and SYBR Premix Ex Taq reagents with an ABI7900 system (Applied Biosystems, Foster City, CA, USA). The primers were designed with Primer Express version 3.0 software and synthesized by Sangon Biotech (Shanghai, China). The primer sequences are listed in Appendix Table 1. Using glyceraldehyde 3-phosphate dehydrogenase (GAPDH) as the internal control, the 2^−ΔΔCt^ method was used to calculate the mRNA expression levels. At least three independent experiments were performed for each reaction.

### DPSC culture

The DPSCs were isolated from rat incisors as described previously (Ge et al., 2013). The DPSCs were cultured in Dulbecco's Modified Eagle's Medium (DMEM) containing 10% fetal bovine serum (FBS), 100 U penicillin, and 100 μg streptomycin (all from Gibco BRL, Grand Island, NY, USA) at 37°C in a humidified atmosphere containing 5% CO_2_.

### siRNA transfection

To explore the significance of the candidate DEGs, we interfered with the expression of the genes using small interfering RNAs (siRNAs) in primary DPSCs. The siRNAs were synthesized by Viewsolid Biotech Co. Ltd. (Beijing, China). Using the Lipofectamine 2000 transfection reagent (Invitrogen, Carlsbad, CA, USA), oligonucleotide transfection was conducted according to the protocol recommended by the manufacturer. A qRT-PCR analysis was used to confirm the knockdown efficiency (Appendix Figure 3A). The siRNA sequences are listed in Appendix Table 2. The cells were collected and used for the cell proliferation and migration assays after transfection for 48 or 96 h, respectively. Non-specific siRNA was used as the negative control (siNC).

### Cell proliferation assay

A Cell Counting Kit 8 (CCK8, Dojindo, kumamoto, Japan) was used to assess the proliferation of the DPSCs. According to the manufacturer's instructions, 2 × 10^3^ cells were seeded into 96-well culture plates and allowed to attach for 24 h. Subsequently, the relevant concentrations of siNC or siRNA were transfected and the cells were incubated for 96 h. At this point, 20 μl CCK-8 solution (5 g/L) was added and the mixture was left for a further 4 h. A Multiscan GO spectrophotometer (Thermo Scientific, Waltham, MA, USA) was used to measure the absorbance at 450 nm.

### Transwell cell migration assay

Cell motility was determined *in vitro* using a Transwell chamber (Costar, Corning, NY, USA). Forty-eight hours after the siNC or siRNA transfection, the cells were trypsinized and placed into the upper wells of the Transwell chamber (40,000 cells per well) in 100 μl serum-free DMEM. In the lower section of the chamber, 600 μl DMEM containing 10% FBS was added. The cells were then cultured in an incubator for 24 h. After the non-migrated cells were scraped off, the membrane was fixed with methanol and the cells were counted after staining with 4′,6-diamidino-2-phenylindole (DAPI; KeyGEN BioTECH, Nanjing, China). The cells in five separate fields were counted using light microscopy at 200× magnification.

### Immunohistochemical (IHC) staining

To examine the protein expression levels of TRPC channel genes, we conducted an IHC analysis using tooth germs at stages E14.5 to P7. The tooth germs were immediately fixed in 4% paraformaldehyde for 24 h at room temperature and the P7 tooth germs were further decalcified in 10% ethylenediaminetetraacetic acid (EDTA) solution (pH 7.4). The specimens were processed for paraffin embedding, and serial 5-μm sections were prepared. All IHC staining was carried out using an SP Kit and a DAB Kit (MXB Biotech, Fujian, China) according to the manufacturer's recommendations. Seven rabbit anti-rat TRPC polyclonal antibody subtypes (Alomone, Jerusalem, Israel) were used after diluting them with water (1:400). In the negative control experiments, rabbit IgG was used to replace the primary antibodies.

### Statistical analysis

Statistical calculations were carried out using SPSS version 20 (IBM Corp., Armonk, NY, USA). Student's *t*-test or one-way analysis of variance (ANOVA) were performed to determine the statistical significance for each comparison; a *P* < 0.05 was considered statistically significant.

## Results

### RNA-Seq analysis

We investigated five stages of tooth germ development (E14.5, E16.5, E18.5, P1, and P7). An unsupervised hierarchal clustering analysis and a principal component analysis (PCA) showed that the tooth germ cells at different stages of development form distinct clusters. The analyses revealed the high degree of similarity among the tooth germ tissues at embryonic stages, and the lower similarity between the embryonic and postnatal tooth germs (Appendix Figures 1A,B).

**Figure 1:**
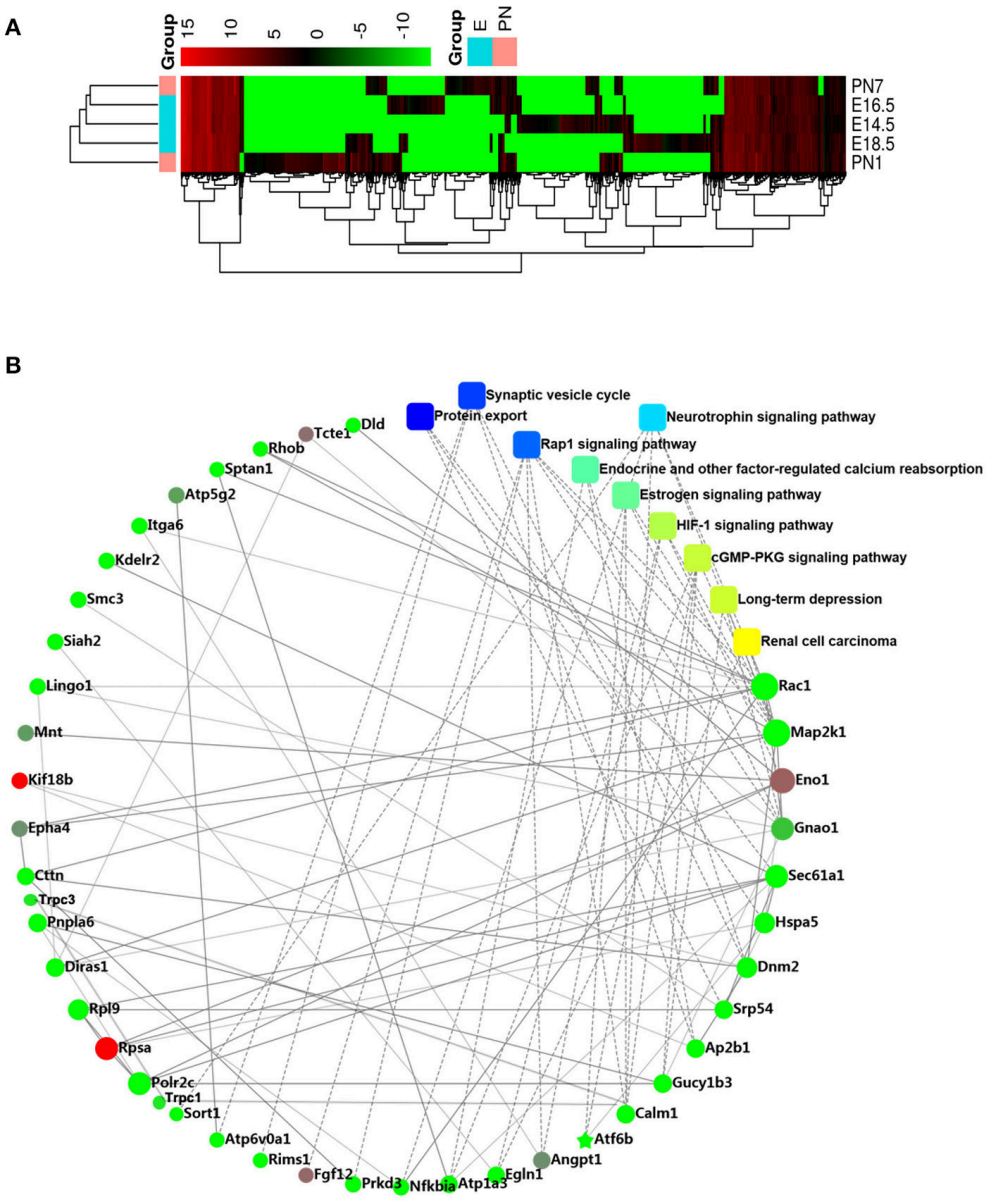
RNA-seq analysis **(A)** Heatmap showes the relative expression patterns of differential genes across all development stages. **(B)** Protein–protein interaction network of the differentially expressed genes (DEGs).

We detected 3368, 3077, 3600, 4441, and 3343 genes in the E14.5, E16.5, E18.5, P1, and P7 stages, respectively, and 1411, 1434, 1536, and 1568 of these genes were detected in the tissues of each pair of adjacent stages, respectively (Appendix Figure 1C). The relative DEG values for the different stages were directly indicated by the heat-map (Figure 1A). We identified 32 significantly up-regulated DEGs and 184 significantly down-regulated DEGs between the embryonic and postnatal stages (false discovery rate (FDR) < 5%, > 2-fold change) (Appendix Table 3).

Based on the PPI network analysis, we discovered several significant DEGs such as *TRPC1, TRPC3, GNAO1, ENO1, EFNB1, CALM1, SIAH2, ATP6V0A1, KDELR2, GTPBP1, POLR2C*, and *SORT1* (Figure 1B). In view of our previous studies on calcium channels (Ju et al., 2015; Gao et al., 2017), we focused on the role of the *TRPC* channel family, including the candidate regulatory genes, *TRPC1* and *TRPC3*. In addition, the GO analysis showed that tooth development is closely related to single-organism cellular processes, intracellular component, binding and protein binding functions (Appendix Figure 2A). The KEGG analysis indicated that tooth development is related to protein export and the synaptic vesicle cycle (Appendix Figure 2B).

### Changes in DEG mRNA levels during rat molar development and effect of DEG knockdown in rat DPSCs

**Figure 2:**
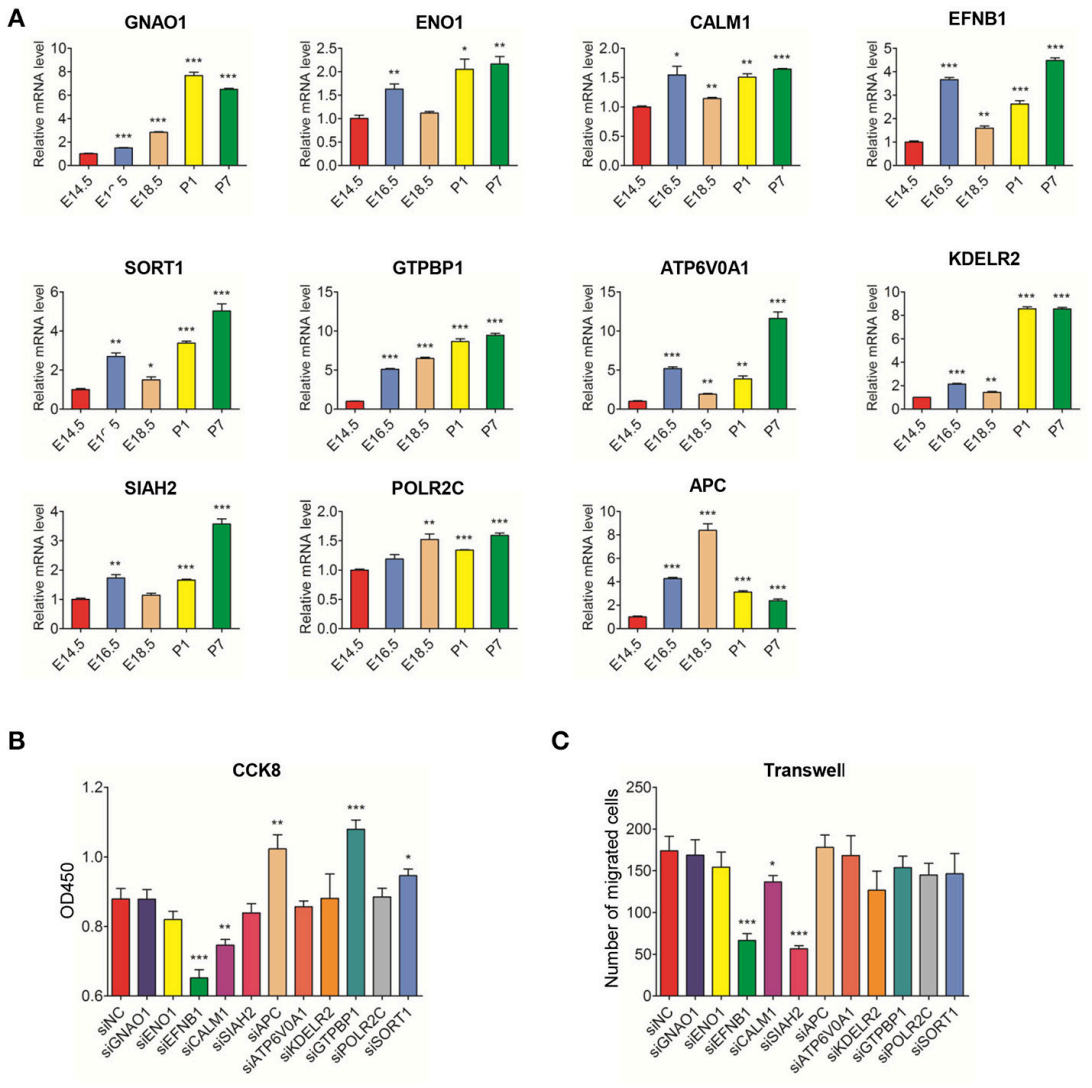
Real-time PCR analysis of DEGs mRNA expression in the development stages of rat tooth germs (E14.5–P7) and the effect of DEG knockdown in rat DPSCs **(A)** Changes in mRNA levels of DEGs during tooth development. Compared with expression at E14.5 stage, the differential expression levels of DEGs at E16.5, E18.5, P1, and P7 stages were evaluated. Data are shown as the mean ± SD; *n* = 3. ^*^p < 0.05, ^***^p < 0.001(Student's *t*-test). **(B)** DPSCs were treated with siNC and siRNA for 96 h and assayed by CCK8. **(C)** DPSCs were treated with siNC and siRNA for 48 h, then the transwell assay was performed. The treated siNC DPSCs was used as a negative control group. The number of migrated cells was displayed as histogram. The data were displayed as the mean value of cells in five fields based on three independent experiments. Results are expressed as the mean ± SD of three independent experiments. ^*^P < 0.05, ^**^P < 0.01, ^***^P < 0.001.

Using qRT-PCR, we studied the expression of candidate DEGs during different developmental stages. The mRNA expression levels of *GNAO1, SORT1, ATP6V0A1, KDELR2*, and *SIAH2* were mainly highly expressed during postnatal stages compared to the E14.5 stage. The expression levels of *ENO1, CALM1, EFNB1, GTPBP1*, and *POLR2C* were significantly higher during the remainder of the developmental process compared to the E14.5 stage (Figure 2A). Based on the qRT-PCR experiments, the expression levels of these genes were slightly different from those indicated by the RNA-Seq data. However, all the data indicated that the candidate genes may be closely related to tooth germ development.

The results of DEG knockdown experiments in DPSCs revealed that proliferation and migration were inhibited in the groups transfected with *EFNB1* and *CALM1* siRNA (compared with siNC group). However, the inhibition of *GTPBP1* and *SORT1* expression promoted DPSC proliferation with no effects on migration. In contrast, the number of migrated cells was notably decreased in the group treated with *SIAH2* siRNA, but there was no impact on the proliferation of DPSCs (Figures 2B,C, Appendix Figure 3B).

### Changes in mRNA levels of TRPC channels during tooth germ development and effect of TRPC channel knockdown in rat DPSCs

**Figure 3:**
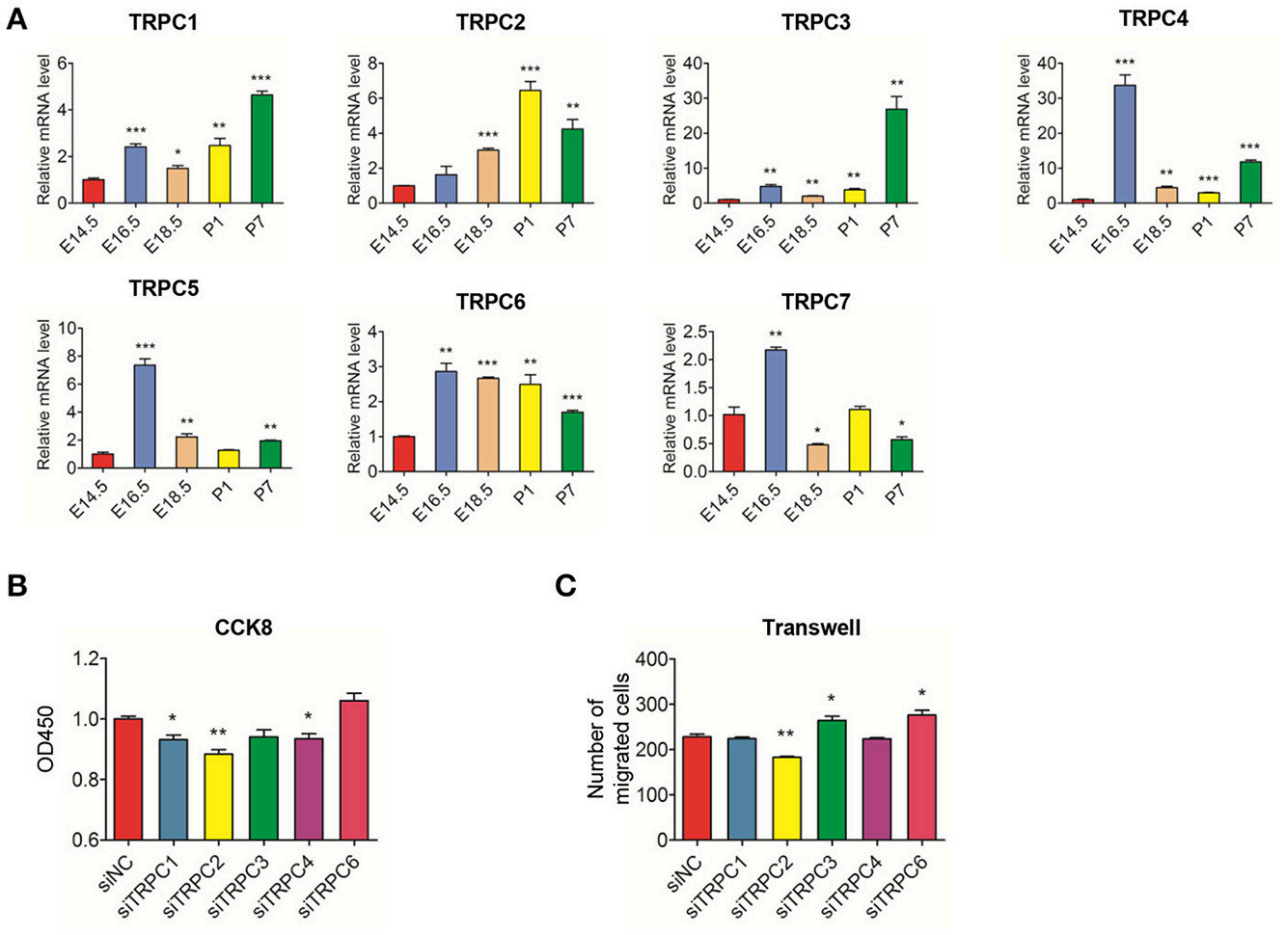
Real-time PCR analysis of TRPC channels mRNA expression in the development stages of rat tooth germs (E14.5–P7) and the effect of knockdown TRPC genes on proliferation and migration of rat DPSCs **(A)** Changes in mRNA levels of TRPC channels during tooth development. Compared with expression at E14.5 stage, the differential expression levels of TRPC channels at E16.5, E18.5, P,1 and P7 stages were evaluated. Data are shown as the mean ± SD; *n* = 3.^*^p < 0.05, ^***^p < 0.001(Student's *t*-test). **(B)** DPSCs were treated with siNC and siTRPC for 96 h and assayed by CCK8. **(C)** DPSCs were treated with siNC and siTRPC for 48 h, then the transwell assay was performed. The treated siNC DPSCs was used as a negative control group. The number of migrated cells was displayed as histogram. The data were displayed as the mean value of cells in five fields from three independent experiments. Results are expressed as the mean ± SD of three independent experiments. ^*^p < 0.05, ^**^p < 0.01.

Using the E14.5 stage as the reference, *TRPC4, TRPC*5, and *TRPC7* mRNA levels were mainly high during the embryonic stage while *TRPC1, TRPC2*, and *TRPC3*, were primarily highly expressed during postnatal stage. The mRNA expression level of *TRPC6* was significantly higher during the remainder of the developmental process compared to the E14.5 stage (Figure 3A). The mRNA expression levels of *TRPC5* and *TRPC7* in DPSCs were too low to be detected. We examined the effects of the remaining *TRPC* family members on the proliferation and migration of DPSCs. Compared with siNC, transfection with *TRPC2* siRNA influenced the proliferation and migration of DPSCs while the DPSCs treated with *TRPC1* and *TRPC4* siRNA only demonstrated inhibited proliferation. The Transwell migration assays revealed that the number of migrated cells was notably decreased in the groups treated with *TRPC3* and *TRPC6* siRNA, but there was no impact on the proliferation of DPSCs in these groups (Figures 3B,C, Appendix Figure 3B).

### IHC staining of TRPC channels at different rat tooth germ developmental stages

**Figure 4:**
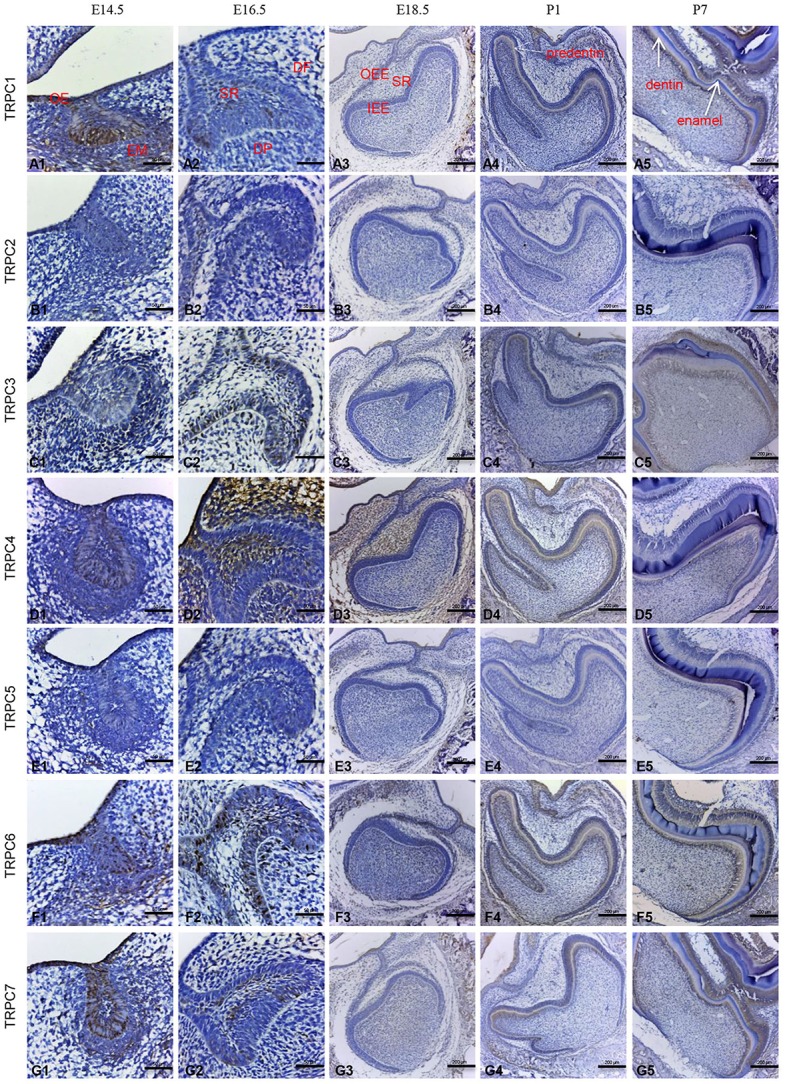
IHC staining of TRPC channels at different rat tooth germdevelopmental stages (E14.5–P7). The TRPC expression was temporally and spatially stage-specific during the tooth germ development. At E14.5, the expression of TRPC1, TRPC3, TRPC6, TRPC7 were mainly observed in epithelial cells **(A1, C1, F1, G1)**. At E16.5, the expression of TRPC1, TRPC3, TRPC6, TRPC7 were observed in outer enamel epithelial cells and inner enamel epithelial cells **(A2, C2, F2, G2)**. The expression of TRPC4 was observed in stellate reticulum cells and dental follicle cells **(D2)**. At E18.5, weak expression of TRPC3, TRPC6, TRPC7 were found in inner and outer enamel epithelial cells **(C3, F3, G3)**. The expression of TRPC4 was observed in stellate reticulum cells and dental follicle cells **(D3)**. At P1, the expression of TRPC1, TRPC3, TRPC4, TRPC6, and TRPC7 were detected in preameloblasts and odontoblasts **(A4, C4, D4, F4, G4)**. At P7, the expression of TRPC1, TRPC3, TRPC6, and TRPC7 were detected in ameloblasts and odontoblasts **(A5, C5, F5, G5)**. TRPC2 and TRPC5 were not detected during the development **(B1–B5, E1–E5)**. Scale bars: 200 μm **(A3–A5)**. Scale bars: 50 μm **(A1, A2)**. OE, oral epithelium; EM, ectoblastic mesenchyme; DF, dental follicle; DP, dental papilla; SR, stellate reticulum; IEE, inner enamel epithelium; OEE, outer enamel epithelium.

**Figure 5:**
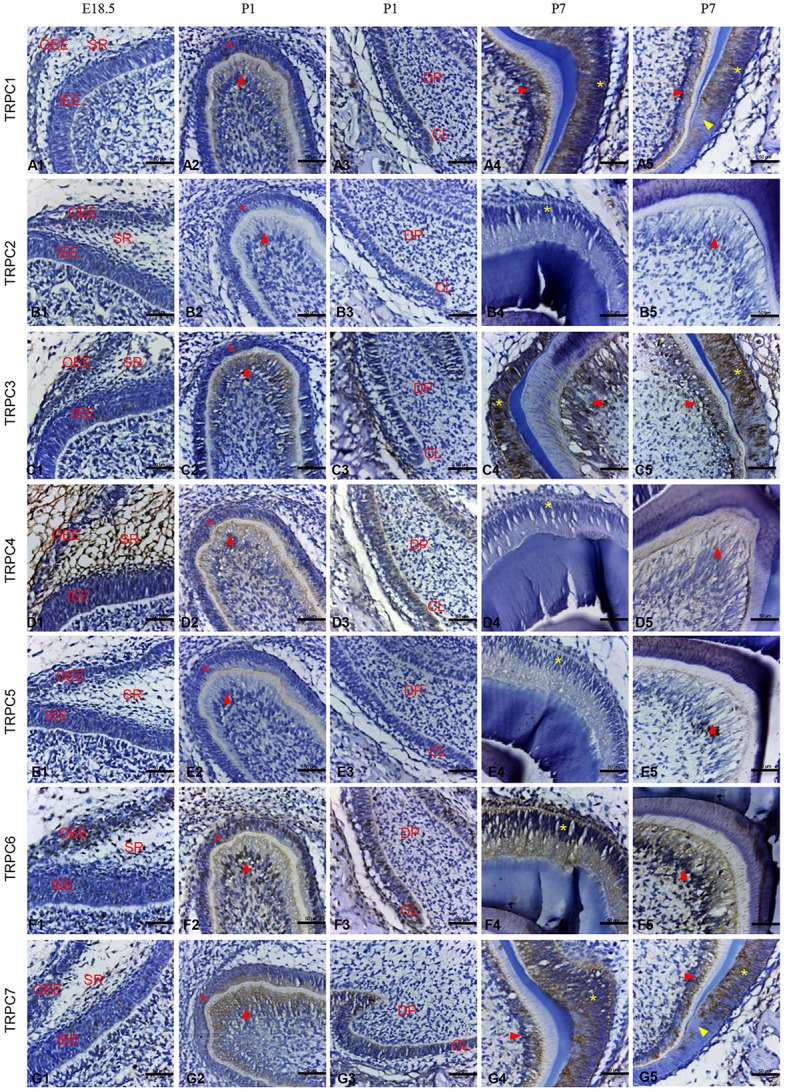
IHC staining of TRPC channels in the rat tooth germ development at E18.5, P1, and P7 stages in higher magnification. At E18.5, weak expression of TRPC3, TRPC6, TRPC7 were found in inner and outer enamel epithelial cells **(C1, F1, G1)**. The expression of TRPC4 was observed in stellate reticulum cells and dental follicle cells **(D1)**. At P1, the expression of TRPC1, TRPC3, TRPC4, TRPC6 and TRPC7 were detected in preameloblasts (^*^ in **A2, C2, D2, F2, G2**), odontoblasts (red arrowhead in **A2, C2, D2, F2, G2**) and cervical loop **(A3, C3, D3, F3, G3)**. At P7, the expression of TRPC1, TRPC3, TRPC6 and TRPC7 were detected in ameloblasts (^*^ in **A4, A5, C4, C5, F4, G4, G5**) and odontoblasts (red arrowhead in **A4, A5, C4, C5, F5, G4, G5**). TRPC1 and TRPC7 were strongly expressed in ameloblasts adjacent to mineralized tissue (yellow arrowhead in **A5, G5**). TRPC2 and TRPC5 were not detected at E18.5, P1 and P7 **(B1–B5, E1–E5)**. Scale bars: 50 μm. OEE, outer enamel epithelium; IEE, inner enamel epithelium; CL, cervical loop.

Given the mRNA expression levels of *TRPC* channels and the significant effects on cell proliferation and migration identified in the knockdown experiments, we further used an IHC analysis to identify the protein expression levels of TRPC channels during development from E14.5 to P7 (Figures 4, 5). The expression position and level of each gene is summarized in Table 1. In the early stage of tooth germ development from bud stage (E14.5) to early bell stage (E18.5), TRPC1, TRPC3, TRPC6 and TRPC7 were mainly detected in epithelial cells and the positive signal gradually decreased, which may have an effect on epithelial cells (Figures 4A1–A3, C1–C3, F1–F3, G1–G3). In contrast, significant expression of TRPC4 was found in the dental follicle and stellate reticulum cells during the early stage, which may be involved cell signal transduction (Figures 4D1–D3, 5D1). In the late postnatal stage, TRPC1, TRPC3, TRPC6, and TRPC7 expression levels were high in ameloblasts and odontoblasts (Figures 4A4–A5, C4–C5, F4–F5, G4–G5, 5A2, A4, C2, C4, F2, F4–F5, G2, G4). This may be closely related to the formation of enamel and dentin. Intriguingly, there were high expression levels of TRPC1 and TRPC7 in ameloblasts, which may be related to enamel mineralization (Figures 5A5, G5). A moderate positive expression level of TRPC4 was only observed in odontoblasts, which may be related to the early formation of dentin (Figure 5D2). TRPC channel expression was absent in the dental papilla during postnatal development (Figures 5A3–G3, A5–G5).

**Table 1:** Localization and immunoreaction intensity of TRPC channels protein during rat molar tooth germ development.

		**TRPC1**	**TRPC2**	**TRPC3**	**TRPC4**	**TRPC5**	**TRPC6**	**TRPC7**
E14.5 Bud stage	dl	+	−	±	−	−	++	++
	de	++	−	±	±	−	++	++
	em	+	−	±	−	−	+	±
E16.5 Cap stage	iee,oee	+	−	++	−	−	++	+
	df,dp	−	−	−	+++	−	+	−
	sr	−	−	−	+++	−	−	−
E18.5 Early bell stage	iee,oee	−	−	+	−	−	+	+
	df,dp	−	−	−	+++	−	−	−
	sr	−	−	−	+++	−	−	−
P1 Late bell stage	pab,ob	+	−	++	++	±	+++	+++
	iee,ds	±	−	+	++	−	++	+
	dp,sr	−	−	−	−	−	−	−
P7 Hard tissue formation stage	ab	+++	−	+++	−	±	+++	+++
	od	+++	−	+++	±	−	+++	+++
	dp	−	−	−	−	−	±	−

*dl, dental lamina; de, dental epithelium; em, ectoblastic mesenchyme; iee, inner enamel epithelium; oee, outer enamel epithelium; df, dental follicle; dp, dental papilla; sr, stellate reticulum; pab, preameloblasts; ob, odontoblasts; ab, ameloblasts. +++, strong; ++, moderate; +, weak; ±, faint; −, negative*.

## Discussion

We completed the RNA sequencing of multiple rat tooth germ samples from different stages of development. As far as we know, this is the first report on the application of RNA-Seq to the study of gene expression profiles in rat tooth germ samples. Compared to DNA microarray experiments, RNA-Seq is associated with a lower background signal and it directly reveals sequence identity, novel transcript isoforms, and unknown genes. Moreover, unlike DNA microarrays, RNA-Seq allows for the detection of genes expressed at extremely low or extremely high levels (Tamm-Rosenstein et al., 2013; Hrdlickova et al., 2017). The advantage of RNA- Seq is that it provides an even more precise measurement of transcriptional levels of gene expressions involved in odontogenesis than all other methods and it therefore helps us to further elucidate the molecular mechanism underlying tooth development.

As part of the bioinformatics analysis, we screened for DEGs associated with rat tooth germ development and found multiple DEGs, including *GNAO1, ENO1, EFNB1, CALM1, SIAH2, ATP6V0A1, KDELR2, GTPBP1, POLR2C*, and *SORT1*. There are few studies on the relationship between the above genes and tooth germ development. Here, we reported for the first time the relationships between a large range of DEGs and tooth germ development and we further present the results of a preliminary functional analysis of the effects of key DEGs on cell proliferation and migration. Among the DEGs, *EFNB1*and *CALM1* had dual effects, as they simultaneously affected cell proliferation and migration. *EFNB1* is essential for the mediation of human skeletal development and bone homeostasis (Nguyen et al., 2016). The extracellular matrix formed by odontoblasts is made in composition as that formed by osteoblasts. *EFNB1* may be related to the maturation of odontoblasts and DPSC odontogenic differentiation. The *CALM1* signaling pathway is believed to have a close connection with precerebellar neuron migration in mice (Kobayashi et al., 2015). We hypothesize that *CALM1* has a close connection with the nerve formation during tooth development. Our follow-up study will focus on the role of these genes in the differentiation of DPSCs in different directions.

In addition, our study showed that TRPC channels participate in the process of tooth germ development. TRPC channels are Ca^2+^-permeable cation channels, and the TRPC channel family contains seven members (TRPC1–TRPC7). Cell function can be influenced by distinct intracellular signals regulated by Ca^2+^ influx via TRPC channels (Ong et al., 2014). TRPC channels have been identified as molecular components of store-operated calcium entry (SOCE) and receptor-operated calcium entry (ROCE) (Parekh and Putney, 2005; Liao et al., 2014). The prominent physiological functions of TRPC channels in tissues derived from ectodermal cells have been demonstrated (Leuner et al., 2011; Sun et al., 2014; Feng et al., 2015). However, there are no reports on the relationship between TRPC channel functions and teeth derived from ectoderm. The protein structures of members of the TRPC channel family are similar as they all contain ankyrin repeat-containing, transient receptor ion channel, and ion transport domains (http://www.ebi.ac.uk/interpro/).

However, according to our results, their transcriptional expression levels vary. To explore the role of TRPC channels during odontogenesis, we investigated the temporal and spatial expression patterns of TRPC channels using IHC analysis. During early tooth germ development from bud stage (E14.5) to early bell stage (E18.5), there were different levels of positive expression in the epithelial cells. These distinguishable distributions of different TRPC channels within the epithelium may be related to the epithelial cell proliferation and differentiation process in tooth germs, (as well as in keratinocytes of the skin and gingiva) (Cai et al., 2005; Leuner et al., 2011). The main functions of the stellate reticulum are to provide mechanical protection during crown formation and nutrient transport from the outlying vascular circulation (Kallenbach, 1980; Ida-Yonemochi et al., 2010). A high expression level of TRPC4 was detected in the stellate reticulum cells. Accordingly, we speculate that TRPC4 might be important for the mechanical protection and nutritional roles of stellate reticulum cells.

The dental crown begins to form in the late bell stage (P1) (Thesleff and Jernvall, 1997). The peripheral cells of the dental papilla differentiate into odontoblasts that secrete dentin, and the inner enamel epithelium turns into ameloblasts that produce enamel (Tompkins, 2006). Enamel and dentin subsequently begin to mineralize to form hard tissue. Phylogenetic studies have revealed that osteoblasts and odontoblasts have many common characteristics (Yang et al., 2014). It has been reported that TRPC channels are expressed in various osteoblast-like cells. In particular, TRPC1 plays an important role in the proliferation of osteoblasts (Abed et al., 2009). High expression levels of TRPC1, TRPC3, TRPC6, and TRPC7 have been detected in odontoblasts. Together with our results, this indicates that TRPC channels may be closely associated with the maturation of odontoblasts. Similar high expression levels have also been found in ameloblasts. Given that the inner enamel epithelium differentiates into ameloblasts, we hypothesize that TRPC channels might be involved in the differentiation of the inner enamel epithelium. Taken together, it suggests that TRPC channels might be more likely related to the acquisition of the differentiated functions. Further studies will be performed on the role of TRPC channels in the differentiational process of dental epithelial cells and mesenchymal cells. In addition, TRPC1 and TRPC7 were expressed to a greater degree in the ameloblasts that were adjacent to the enamel tissue associated with mineralization than the normal ameloblasts. This indicates that these two channels might have more influence on enamel mineralization than the other channels, and it will provide inspiration for our future research.

We also examined the effect of inhibiting the expression of TRPC genes on DPSC proliferation and migration. DPSCs are a part of the ectomesenchyme, which is derived from neural crest cells (Janebodin et al., 2011; Arakaki et al., 2012). All members of the TRPC family have been identified in neurons in recent studies and signals mediated by Ca^2+^ influxes via TRPC channels have been reported to be connected with neuron proliferation and differentiation (Sun et al., 2014). Based on this, together with our results, we speculate that Ca^2+^ influx regulated by TRPC channels might be an important factor affecting the growth and migration of DPSCs. In view of the potentially prominent effect of TRPC channels on the proliferation and migration of DPSCs, we hypothesize that they might also influence the differentiation of DPSCs. Taking into account the IHC staining results, we plan to study the roles of TRPC channels in the odontogenic and neural differentiation of DPSCs.

In summary, a transcriptional gene expression analysis was conducted and a number of DEGs were identified that might be functionally related to rat tooth germ development. Our data provide important information on novel genes involved in odontogenesis, which could be further explored by studying tooth formation in genetically engineered mice. Our findings may help to shed light on the molecular mechanism underlying tooth germ development and provide a theoretical basis for future research on the expression and function of genes involved in human tooth development and regeneration.

## Author contributions

JY: conception and design, collection and assembly of data, and data analysis and manuscript writing; WC and XL: data analysis and interpretation; SL and SZ: conception and design, manuscript editing, and final approval of manuscript.

### Conflict of interest statement

The authors declare that the research was conducted in the absence of any commercial or financial relationships that could be construed as a potential conflict of interest.
