# Supplementary material for: RNA-Sequencing Analyses Demonstrate the Involvement of Canonical Transient Receptor Potential Channels in Rat Tooth Germ Development
Source: Front Physiol. 2017 Jun 29;8:455. doi: 10.3389/fphys.2017.00455 (PMC5489664; doi:10.3389/fphys.2017.00455)
Supplement: Supplementary file 2 [file DataSheet1.DOCX]

RNA-Sequencing Analyses Demonstrate the Involvement of Canonical Transient Receptor Potential Channels in Rat Tooth Germ Development

Jun Yang, Wenping Cai, Xi Lu, Shangfeng Liu^*^, Shouliang Zhao^*^

**Appendix figure1. The validation of the RNAseq analysis and the number of expressed genes in different development stages**

(A) Cluster dendrogram;

(B) Principal component analysis for five stages of tooth germs;

(C) The number of expressed genes in different rat tooth germ development stages.

**Appendix figure2.** **RNA-seq analysis**

(A) GO analysis;

(B) KEGG analysis.

**Appendix figure3.** **siRNA interference efficiency and dapi staining of transwell assay**

(A) siRNA interference efficiency of differentially expressed genes;

(B) Representative phase contrast image of dapi staining in transwell assay.

**Appendix Table 1. The sequences of gene primers**

**Appendix Table 2. siRNA sequences**

**Appendix Table 3. List of the main genes corresponding to the heatmap**

**Appendix Table 1**

| **Gene** | **Primer sequence** |
| --- | --- |
| TRPC1 | Forward TTCCTTCACGATTGGACTGA |
|  | Reverse CAATGAACGAGTGGAAGGTG |
| TRPC2 | Forward ATCGTCATGGTGATTGTGCT |
|  | Reverse AGCATCGTCCTCGATCTTCT |
| TRPC3 | Forward TGAGGTGAACGAAGGTGAAC |
|  | Reverse ATCAAGATGGCCAGTTCCTC |
| TRPC4 | Forward CGTCATCTCTCTGGTTGTCC |
|  | Reverse TGGTCGGCAATTAGTTGGTA |
| TRPC5 | Forward TGCCTTGGGTTCTAGGTTTC |
|  | Reverse CCATCAAATTCCACCAATCA |
| TRPC6 | Forward ATCTGGCCAGGATAAAGTGG |
|  | Reverse AATCTGCAGAGGTCCAAAGC |
| TRPC7 | Forward TCCCTGCTCCCTTTAACCTG |
|  | Reverse GCCCATTTCCAGGTCGTTTT |
| APC | Forward TTTGAGTTCTAGCGGCACAC |
|  | Reverse ACACCATTTCCACCTTCGTT |
| GNAO1 | Forward AAAGACCTCTTTGGCGAGAA |
|  | Reverse GTGTTGGAGCCTGGGTATTC |
| ENO1 | Forward GGGGAAGGGTGTCTCAAAGG |
|  | Reverse GTGCCGTCCATCTCGATCAT |
| CALM1 | Forward GCGAAGAAGAAATCCGTGAG |
|  | Reverse GTTCTGCCGCACTGATGTAG |
| EFNB1 | Forward CGTGTCCTGGAGCTCTCTTA |
|  | Reverse TCCAATCTTCGGGTAGATCA |
| SORT1 | Forward AGTCATCGCTCACGGTAGTG |
|  | Reverse ATGCGGTCCTTCTAGCATCT |
| GTPBP1 | Forward GTCTGCGCACAGGAGATAAA |
|  | Reverse GTCTGGAGGAGCTTGGTGAT |
| ATP6V0A1 | Forward TGTTCAGCGGCCGATACATT |
|  | Reverse CTCCTCCGTCCAGTTCCCTA |
| KDELR2 | Forward TGATGGAAATCACGACACCT |
|  | Reverse GGTCCACAAGATCTCAAGAGG |
| SIAH2 | Forward AGCATCAGGAACCTGGCTAT |
|  | Reverse TGGTGGCATACTTACAGGGA |
| POLR2C | Forward TGCCCATAATAGCCATTGAC |
|  | Reverse GGAGTACTGCAGCTTGTCCA |

**Appendix Table 2**

| **Gene** | **sense（5'-3'）** | **antisense（5'-3'）** |
| --- | --- | --- |
| TRPC1 | CCCACCUGUAAGAAGAUAATT | UUAUCUUCUUACAGGUGGGTT |
|  | CCAGCUGAUAGCAAAUCAUTT | AUGAUUUGCUAUCAGCUGGTT |
|  | GCUCAGCUUUGUUAUGAAUTT | AUUCAUAACAAAGCUGAGCTT |
| TRPC2 | GCCUUCUUUGACUCAUCAATT | UUGAUGAGUCAAAGAAGGCTT |
|  | GCCGAUAUGACCUGCUGAATT | UUCAGCAGGUCAUAUCGGCTT |
|  | GCAAGUGCUGUCUUCCAUUTT | AAUGGAAGACAGCACUUGCTT |
| TRPC3 | GCUGCGCAUUGCCAUAAAUTT | AUUUAUGGCAAUGCGCAGCTT |
|  | CCAUCUUCAUUGCUGCCUUTT | AAGGCAGCAAUGAAGAUGGTT |
|  | CCCUCAGAUCAUAUCGGAATT | UUCCGAUAUGAUCUGAGGGTT |
| TRPC4 | CCAGAGAGCUGGAAAUCAUTT | AUGAUUUCCAGCUCUCUGGTT |
|  | CCUUGUAUCUGGCGACAAUTT | AUUGUCGCCAGAUACAAGGTT |
|  | GCUGAUCGCUAUGAUGAAUTT | AUUCAUCAUAGCGAUCAGCTT |
| TRPC5 | GGUAGGAGCUGUGGAACUUTT | AAGUUCCACAGCUCCUACCTT |
|  | CCCGAGAACUGGAGAUCAUTT | AUGAUCUCCAGUUCUCGGGTT |
|  | CCUUGCGUCUCAUAUCCUUTT | AAGGAUAUGAGACGCAAGGTT |
| TRPC6 | GCUGCUCAUUGCCAGGAAUTT | AUUCCUGGCAAUGAGCAGCTT |
|  | GCUUGCCAACAUUGAGAAATT | UUUCUCAAUGUUGGCAAGCTT |
|  | GCUCCUCUCCAUAUGGUAUTT | AUACCAUAUGGAGAGGAGCTT |
| TRPC7 | CCAAGACCCUCAAUUUCAATT | UUGAAAUUGAGGGUCUUGGTT |
|  | GCUCCCGUAUGAAUGCCUATT | UAGGCAUUCAUACGGGAGCTT |
|  | GCAUCAAACUUGCCAUUAATT | UUAAUGGCAAGUUUGAUGCTT |
| APC | GCCCUUUAUACAGAAAGAUTT | AUCUUUCUGUAUAAAGGGCTT |
|  | GAGCGCCUUAAAGAAUUUATT | UAAAUUCUUUAAGGCGCUCTT |
|  | GGGAAAUCCAAGCAUUAAATT | UUUAAUGCUUGGAUUUCCCTT |
| GNAO1 | GCGUGGAGUAUGGUGACAATT | UUGUCACCAUACUCCACGCTT |
|  | CCUCCACUUCAGGCUGUUUTT | AAACAGCCUGAAGUGGAGGTT |
|  | GCAUGCACGAGUCUCUCAUTT | AUGAGAGACUCGUGCAUGCTT |
| ENO1 | GGACGGCACAGAGAAUAAATT | UUUAUUCUCUGUGCCGUCCTT |
|  | GGCACAGAGAAUAAAUCUATT | UAGAUUUAUUCUCUGUGCCTT |
|  | CUCCGAGACAAUGAUAAGATT | UCUUAUCAUUGUCUCGGAGTT |
| CALM1 | CACAGAUAGCGAAGAAGAATT | UUCUUCUUCGCUAUCUGUGTT |
|  | CCAUCACAACAAAGGAGCUTT | AGCUCCUUUGUUGUGAUGGTT |
|  | GGAAAAGCUAACAGAUGAATT | UUCAUCUGUUAGCUUUUCCTT |
| EFNB1 | GCAAGUGGCUUGUGGCUAUTT | AUAGCCACAAGCCACUUGCTT |
|  | GCACUGUGCUUGAUCCCAATT | UUGGGAUCAAGCACAGUGCTT |
|  | GCGUCAUCUUCCUGCUCAUTT | AUGAGCAGGAAGAUGACGCTT |
| SORT1 | GGAAGAGUGUUCAGGUCAUTT | AUGACCUGAACACUCUUCCTT |
|  | GCAGUAUGUUUGGCCAAAUTT | AUUUGGCCAAACAUACUGCTT |
|  | CCACGUGUCAACAGACCAATT | UUGGUCUGUUGACACGUGGTT |
| GTPBP1 | GCAGUAUGACAGUCUACUUTT | AAGUAGACUGUCAUACUGCTT |
|  | GGAAACGAGUCGGAGACAATT | UUGUCUCCGACUCGUUUCCTT |
|  | GCAGCCUUGAGUGGACAAATT | UUUGUCCACUCAAGGCUGCTT |
| ATP6V0A1 | CCAGGGACAUGAUCGACUUTT | AAGUCGAUCAUGUCCCUGGTT |
|  | CCCUGAACCUCUGCAACAUTT | AUGUUGCAGAGGUUCAGGGTT |
|  | GCACUUACCGAGAAAUUAATT | UUAAUUUCUCGGUAAGUGCTT |
| KDELR2 | CCUGAUCUACAUGAAGUUUTT | AAACUUCAUGUAGAUCAGGTT |
|  | GCCUCUCAUUCCUAGUCAATT | UUGACUAGGAAUGAGAGGCTT |
|  | GGAUCUGGCGCUUCUACUUTT | AAGUAGAAGCGCCAGAUCCTT |
| SIAH2 | GCACCUGGUGUGUAACCAATT | UUGGUUACACACCAGGUGCTT |
|  | GCAUCAGGAACCUGGCUAUTT | AUAGCCAGGUUCCUGAUGCTT |
|  | GCAAGCAAGCAGAGAACUUTT | AAGUUCUCUGCUUGCUUGCTT |
| POLR2C | GCGCACAGGCUUGGUUUAATT | UUAAACCAAGCCUGUGCGCTT |
|  | CCCGAGAUCUCAUCUCCAATT | UUGGAGAUGAGAUCUCGGGTT |
|  | GCUGAGACUUCGAGCCUAUTT | AUAGGCUCGAAGUCUCAGCTT |
| siNC | UUCUCCGAACGUGUCACGUTT | ACGUGACACGUUCGGAGAATT |

**Appendix Table 3**

| up-regulated | Log2Foldchange | pvalue |
| --- | --- | --- |
| ZNF703 | 16.22 | 2.19E-07 |
| PHOX2A | 16.86 | 2.57E-04 |
| SIX4 | 15.71 | 1.60E-06 |
| ITGB1BP2 | 16.57 | 1.74E-04 |
| WBP2 | 16.39 | 1.61E-03 |
| REPIN1 | 16.65 | 1.57E-05 |
| SNORA62 | 17.9 | 1.88E-05 |
| KIF18B | 14.4 | 3.83E-05 |
| TCTE1 | 1.14 | 1.18E-02 |
| POU5F2 | 18.31 | 7.43E-06 |
| GATA2 | 16.24 | 3.91E-05 |
| STRN4 | 1.73 | 4.26E-04 |
| CKB | 18.02 | 3.11E-04 |
| COX6A1 | 17.32 | 1.60E-05 |
| FAM172A | 18.31 | 7.43E-06 |
| GATC | 17.32 | 1.60E-05 |
| STOX2 | 14.88 | 2.35E-05 |
| XXbac-BPG252P9.9 | 17.41 | 3.01E-05 |
| CCT6A | 16.6 | 3.65E-04 |
| ENO1 | 2.47 | 2.84E-02 |
| RPSA | 17.9 | 1.88E-05 |
| ITM2B | 16.57 | 2.18E-04 |
| RPL38 | 18.91 | 3.22E-05 |
| FAM109A | 14.96 | 1.37E-05 |
| ZNF775 | 16.65 | 1.57E-05 |
| MIR2277 | 18.31 | 7.43E-06 |
| SNORA6 | 17.9 | 1.88E-05 |
| FGF12 | 1.71 | 3.77E-03 |
| AL021546.6 | 17.32 | 1.60E-05 |
| MED19 | 1.62 | 2.12E-02 |
| INTS1 | 13.8 | 1.56E-04 |
| CORO2A | 13.75 | 2.38E-06 |
|  |  |  |
| down-regulated | Log2Foldchange | pvalue |
| ITGA6 | -14.15 | 4.46E-05 |
| ATP1A3 | -15.52 | 5.02E-04 |
| IFT80 | -14.5 | 1.76E-05 |
| PCK2 | -15.52 | 2.73E-06 |
| SIX2 | -17.16 | 3.53E-05 |
| NRXN3 | -16.11 | 4.37E-03 |
| STAC3 | -1.27 | 1.82E-02 |
| TKT | -16.5 | 3.90E-04 |
| DLD | -16.7 | 5.05E-04 |
| SEPN1 | -18.22 | 4.83E-04 |
| IGFBP4 | -16.24 | 2.03E-06 |
| KTN1 | -15.71 | 1.13E-03 |
| RAC1 | -16.27 | 1.72E-04 |
| RRP7A | -14.35 | 2.94E-06 |
| CALM2 | -21.15 | 3.26E-03 |
| BRD7 | -18.31 | 2.44E-04 |
| BRD3 | -16.58 | 5.10E-06 |
| ANGEL1 | -14.23 | 3.01E-06 |
| TAX1BP1 | -15.77 | 8.38E-05 |
| SEC61A1 | -15.58 | 8.15E-06 |
| SIAH2 | -19.27 | 2.49E-05 |
| CDRT1 | -16.48 | 4.72E-06 |
| SORT1 | -13.92 | 8.08E-06 |
| RP11-432B6.3 | -14.5 | 1.76E-05 |
| CSTF2T | -14.23 | 3.01E-06 |
| ANGPT1 | -1.22 | 4.73E-02 |
| SRRT | -1.36 | 2.92E-02 |
| ATP6V0A1 | -18.9 | 2.90E-04 |
| RPL36A-HNRNPH2 | -18.15 | 5.42E-06 |
| NRON | -19.45 | 2.97E-06 |
| SNORA51 | -16.7 | 1.60E-06 |
| GSTP1 | -17.58 | 1.60E-06 |
| RP11-972K6.1 | -2.65 | 3.93E-02 |
| TRIM16 | -16.48 | 4.72E-06 |
| NSG2 | -16.59 | 2.59E-06 |
| KIAA0195 | -15.86 | 9.45E-04 |
| GRIK1 | -15.22 | 4.36E-05 |
| PNPLA6 | -15.82 | 6.32E-04 |
| FRMD4A | -16.17 | 1.09E-03 |
| COQ9 | -17.58 | 4.79E-06 |
| SNORD57 | -16.7 | 1.60E-06 |
| FOXK1 | -12.74 | 4.20E-06 |
| BCAP31 | -17.28 | 6.48E-04 |
| RPL36A | -18.15 | 5.42E-06 |
| HIPK2 | -1.38 | 3.25E-02 |
| RP11-466C23.4 | -13.77 | 3.32E-06 |
| YAF2 | -19.03 | 4.67E-04 |
| SAP130 | -17.66 | 1.67E-04 |
| SMC3 | -15.63 | 9.86E-04 |
| BANF1 | -17.5 | 8.29E-06 |
| PLEKHO1 | -17.48 | 2.27E-06 |
| REV3L | -1.32 | 1.17E-02 |
| ANXA5 | -16.91 | 6.79E-05 |
| RUVBL1 | -16.02 | 4.16E-07 |
| DDX56 | -16.85 | 2.97E-05 |
| SHD | -17.79 | 2.17E-03 |
| MIR939 | -15.68 | 1.35E-03 |
| CALD1 | -16.74 | 3.62E-04 |
| TBKBP1 | -16.24 | 2.25E-03 |
| XRCC1 | -17.19 | 5.38E-05 |
| PHF6 | -15.41 | 8.31E-04 |
| SNORD86 | -16.7 | 1.60E-06 |
| C11orf95 | -13.77 | 3.32E-06 |
| L34079.2 | -17.19 | 5.38E-05 |
| STC1 | -1.65 | 3.91E-02 |
| RP11-385D13.1 | -16.48 | 4.72E-06 |
| AMOT | -16.51 | 9.64E-04 |
| MCOLN1 | -15.82 | 6.32E-04 |
| PTPRK | -16.54 | 4.45E-06 |
| IRGQ | -17.19 | 5.38E-05 |
| NUS1 | -14.04 | 3.14E-06 |
| C21orf91-OT1 | -18.36 | 1.61E-06 |
| CPSF1 | -15.68 | 1.35E-03 |
| MIR1292 | -16.7 | 1.60E-06 |
| CARM1 | -16.13 | 6.79E-04 |
| AC006538.4 | -16.28 | 1.10E-03 |
| BUD31 | -18.09 | 5.41E-04 |
| RAB39A | -15.51 | 2.33E-06 |
| C5orf51 | -13.86 | 3.26E-06 |
| HSPA5 | -16.66 | 3.50E-05 |
| PISD | -16.38 | 2.83E-04 |
| ARF5 | -17.81 | 1.77E-06 |
| PPP2R5D | -18.36 | 7.91E-06 |
| CA10 | -18.23 | 5.42E-05 |
| CTD-2545M3.6 | -15.54 | 4.46E-04 |
| SHISA9 | -15.84 | 5.49E-06 |
| MIR5010 | -18.9 | 2.90E-04 |
| TRIM59 | -14.5 | 1.76E-05 |
| RAB14 | -15.51 | 8.18E-04 |
| R3HDM2 | -1.27 | 1.82E-02 |
| GNAO1 | -5.37 | 2.21E-02 |
| FSCN3 | -17.81 | 1.77E-06 |
| MNT | -2.26 | 1.72E-02 |
| POLR2C | -17.58 | 4.79E-06 |
| RHOB | -16.59 | 9.82E-04 |
| RPL9 | -17.71 | 1.56E-06 |
| TMCO1 | -17.39 | 2.08E-05 |
| PEPD | -16.08 | 4.81E-05 |
| BSN | -13.01 | 4.15E-04 |
| RIMS1 | -15.53 | 1.10E-06 |
| MIR1234 | -15.68 | 1.35E-03 |
| NUP37 | -18.84 | 1.30E-06 |
| DYM | -17.12 | 1.60E-03 |
| OSTM1 | -16.81 | 1.49E-03 |
| SNORD56 | -16.7 | 1.60E-06 |
| LRRFIP2 | -16.1 | 1.39E-03 |
| HNRNPH2 | -18.15 | 5.42E-06 |
| RPS2P46 | -15.79 | 2.21E-06 |
| PFKFB2 | -15.6 | 4.31E-06 |
| C2orf61 | -21.15 | 3.26E-03 |
| JADE3 | -15.33 | 8.47E-04 |
| SNORD110 | -16.7 | 1.60E-06 |
| BTBD11 | -1.37 | 7.45E-03 |
| STX17 | -17.26 | 4.29E-05 |
| CTD-2207O23.10 | -15.82 | 6.32E-04 |
| ZNF217 | -13.76 | 3.33E-06 |
| STMN2 | -19.28 | 9.22E-03 |
| NFKBIA | -16.62 | 1.33E-04 |
| RP11-761B3.1 | -21.15 | 3.26E-03 |
| PAQR8 | -14.04 | 3.13E-06 |
| RP1-317E23.6 | -18.22 | 4.83E-04 |
| AC100787.1 | -15.86 | 9.45E-04 |
| SLITRK2 | -14.96 | 4.08E-05 |
| CTTN | -15.32 | 3.72E-05 |
| SLC39A3 | -16.28 | 1.10E-03 |
| SLC39A6 | -16.93 | 1.79E-06 |
| EGLN1 | -16.16 | 5.27E-03 |
| SPIB | -15.54 | 4.46E-04 |
| C21orf91 | -18.36 | 1.61E-06 |
| AC067852.1 | -18.9 | 2.90E-04 |
| USP25 | -16.08 | 4.36E-05 |
| ADAMTS6 | -1.79 | 4.37E-02 |
| LDB2 | -1.98 | 3.17E-02 |
| PAFAH1B2 | -18.46 | 7.53E-04 |
| ARMCX4 | -13.99 | 2.69E-05 |
| SLC35C2 | -17.31 | 3.19E-07 |
| NOP56 | -16.7 | 1.60E-06 |
| MAPRE2 | -15.87 | 1.40E-03 |
| POM121 | -14.63 | 4.13E-11 |
| SENP2 | -14.5 | 5.23E-05 |
| AC009052.12 | -17.58 | 4.79E-06 |
| MIR4329 | -16.51 | 9.64E-04 |
| ATF6B | -12.5 | 4.44E-06 |
| KDELR2 | -18.89 | 5.78E-07 |
| TNXB | -12.5 | 4.44E-06 |
| EPHA4 | -1.3 | 2.37E-02 |
| PVRL1 | -13.7 | 3.37E-06 |
| SLC6A9 | -1.05 | 1.57E-02 |
| DIRAS1 | -16.28 | 1.10E-03 |
| OPCML | -15.99 | 1.05E-02 |
| PRKD3 | -16.43 | 4.52E-04 |
| DCTN3 | -18.68 | 6.42E-04 |
| NTM | -16.36 | 7.49E-05 |
| SPTAN1 | -13.26 | 3.72E-06 |
| GUCY1B3 | -15.4 | 2.38E-06 |
| GBF1 | -2.03 | 3.04E-02 |
| RPL17P42 | -15.13 | 1.26E-03 |
| COL5A2 | -15.44 | 2.58E-03 |
| HMGB1P5 | -18.9 | 5.57E-03 |
| MTFR1L | -18.22 | 4.83E-04 |
| CERS2 | -16.83 | 4.89E-04 |
| LINGO1 | -16.63 | 1.42E-03 |
| EML4 | -15.76 | 1.60E-03 |
| CHRNB4 | -15.97 | 1.10E-06 |
| BTAF1 | -14.37 | 5.27E-04 |
| KIAA1432 | -16.22 | 1.66E-03 |
| MAP2K1 | -16.42 | 5.01E-06 |
| ATP5G2 | -2.65 | 3.93E-02 |
| SRP54 | -16.1 | 2.86E-04 |
| MMP24 | -14.12 | 3.08E-06 |
| RP11-400F19.18 | -18.9 | 2.90E-04 |
| FAM19A5 | -15.83 | 2.19E-06 |
| DAGLB | -18.89 | 5.78E-07 |
| DNM2 | -16.53 | 6.40E-04 |
| RP11-107I14.5 | -18.43 | 5.25E-05 |
| CTNNBL1 | -16.67 | 6.00E-04 |
| FAM8A1 | -14.04 | 3.14E-06 |
| AP000350.4 | -15.87 | 2.21E-06 |
| POLD1 | -15.54 | 4.46E-04 |
| STAU1 | -16.1 | 9.83E-04 |
| ID2 | -17.79 | 1.27E-03 |
| WIZ | -14.56 | 2.81E-06 |
| HP1BP3 | -18.12 | 1.98E-04 |
| AP2B1 | -15.13 | 1.26E-03 |
