# Supplementary figures and images for: RNA-Sequencing Analyses Demonstrate the Involvement of Canonical Transient Receptor Potential Channels in Rat Tooth Germ Development
Source: Front Physiol. 2017 Jun 29;8:455. doi: 10.3389/fphys.2017.00455 (PMC5489664; doi:10.3389/fphys.2017.00455)

# Appendix Figure 1

A

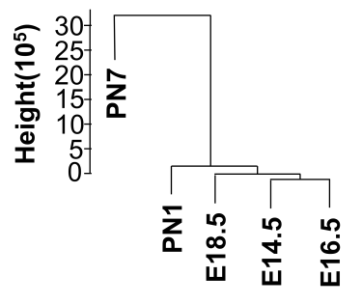

B

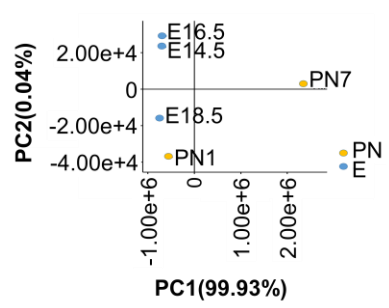

C

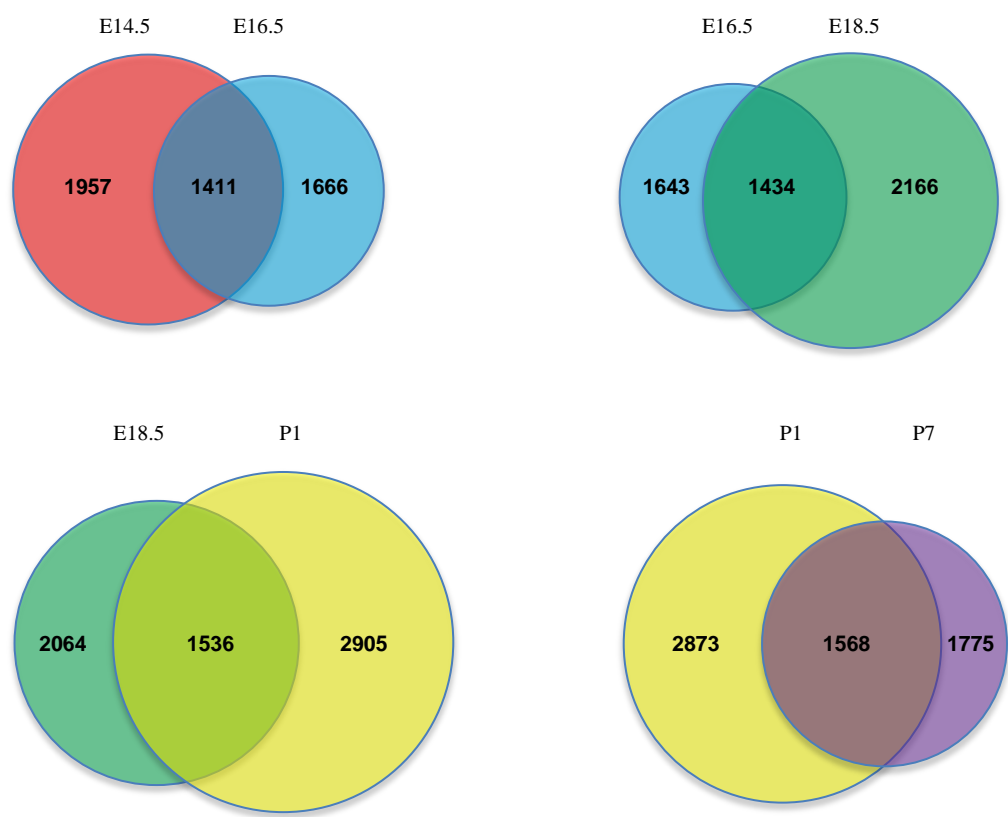

Appendix figure 2

A

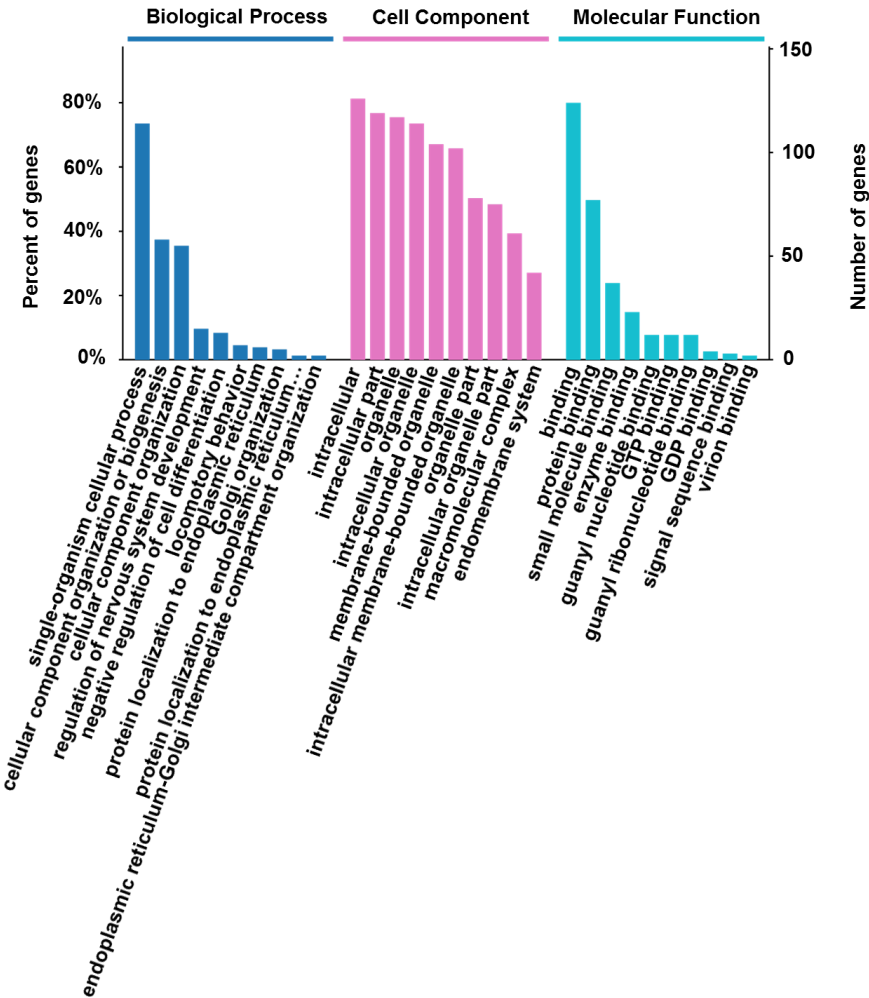

B

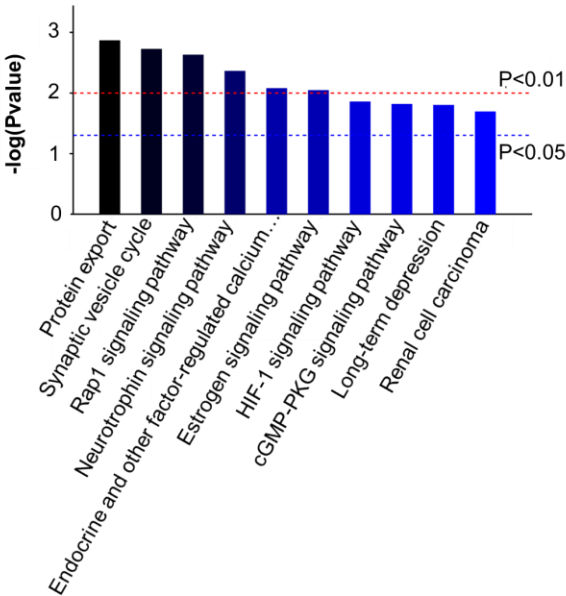

# Appendix Figure 3

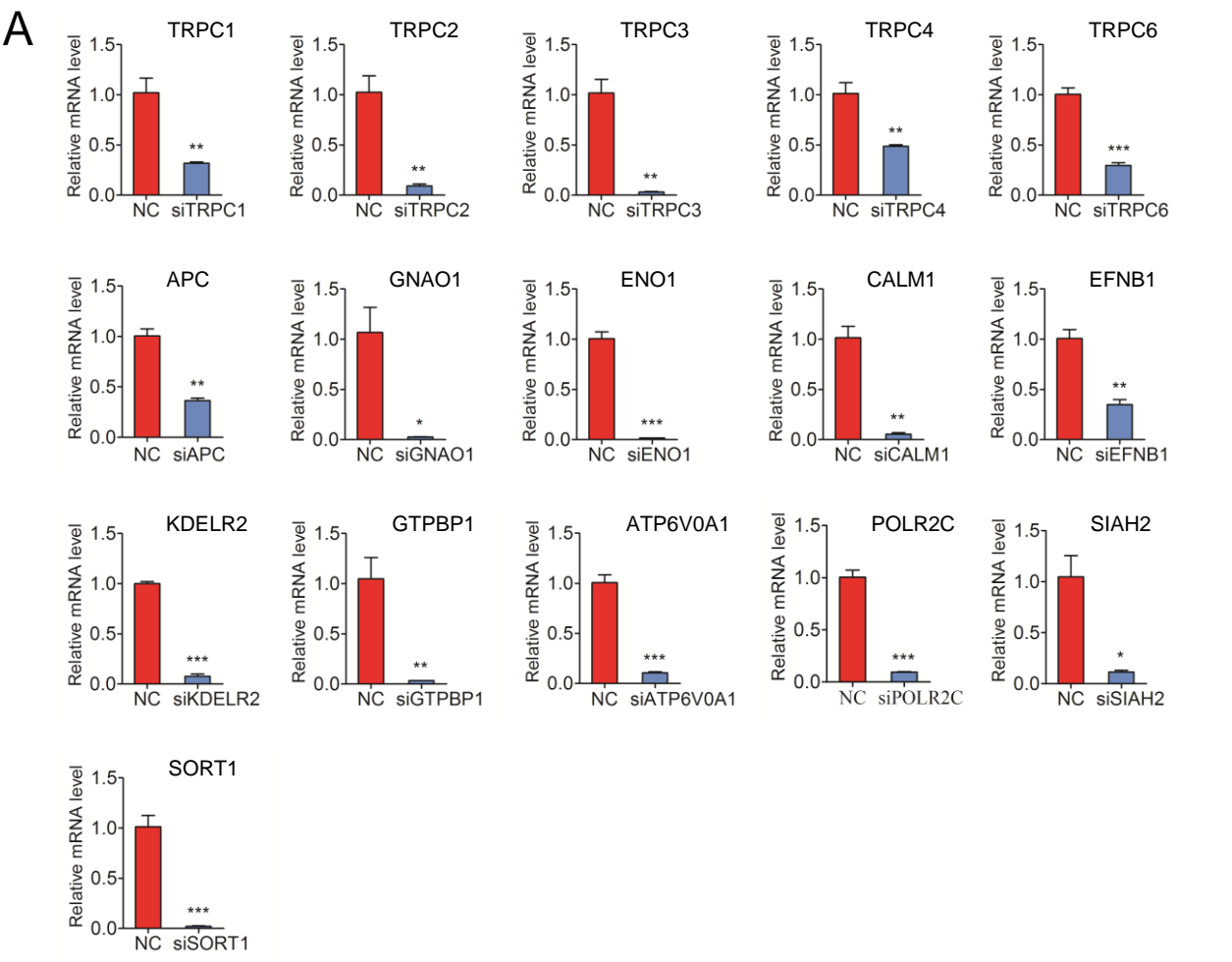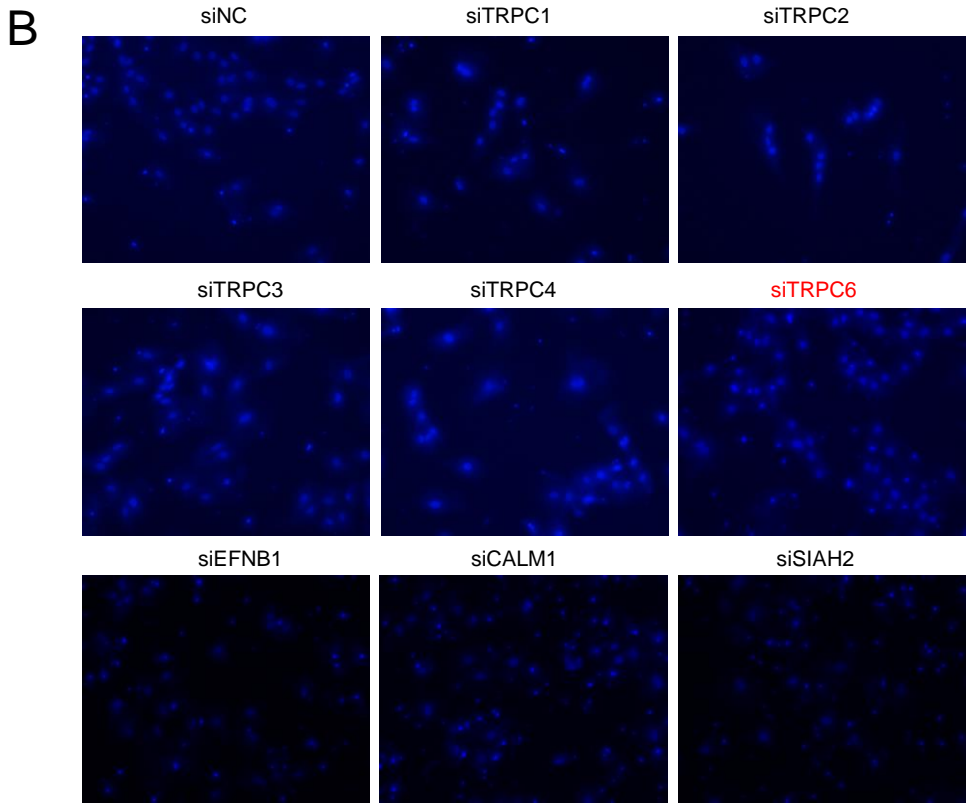

Supplement: Supplementary file 3 [file Image1.pdf]
